# On cognitive ecology and the environmental factors that promote Alzheimer disease: lessons from *Octodon degus* (Rodentia: Octodontidae)

**DOI:** 10.1186/s40659-016-0074-7

**Published:** 2016-02-20

**Authors:** Daniela S. Rivera, Nibaldo C. Inestrosa, Francisco Bozinovic

**Affiliations:** Departamento de Ecología and Center of Applied Ecology and Sustainability (CAPES), Facultad de Ciencias Biológicas, Pontificia Universidad Católica de Chile, 6513677 Santiago, Chile; Centro de Envejecimiento y Regeneración (CARE), Departamento de Biología Celular y Molecular, Facultad de Ciencias Biológicas, Pontificia Universidad Católica de Chile, 6513677 Santiago, Chile; Centro UC Síndrome de Down, Pontificia Universidad Católica de Chile, Santiago, Chile; Centro de Excelencia en Biomedicina de Magallanes (CEBIMA), Universidad de Magallanes, Punta Arenas, Chile; Departamento de Ciencias Ecológicas, Facultad de Ciencias Biológicas, Pontificia Universidad Católica de Chile, Casilla 114-D, Avenida Libertador Bernardo O’Higgins 340, Santiago, Chile

**Keywords:** Cognitive ecology, *Octodon degus*, Social behavior, Stress, Aging, Alzheimer’s disease

## Abstract

Cognitive ecologist posits that the more efficiently an animal uses information from the biotic and abiotic environment, the more adaptive are its cognitive abilities. Nevertheless, this approach does not test for natural neurodegenerative processes under field or experimental conditions, which may recover animals information processing and decision making and may explain, mechanistically, maladaptive behaviors. Here, we call for integrative approaches to explain the relationship between ultimate and proximate mechanisms behind social behavior. We highlight the importance of using the endemic caviomorph rodent *Octodon degus* as a valuable natural model for mechanistic studies of social behavior and to explain how physical environments can shape social experiences that might influence impaired cognitive abilities and the onset and progression of neurodegenerative disorders such as Alzheimer disease. We consequently suggest neuroecological approaches to examine how key elements of the environment may affect neural and cognitive mechanisms associated with learning, memory processes and brain structures involved in social behavior. We propose the following three core objectives of a program comprising interdisciplinary research in *O. degus*, namely: (1) to determine whether diet types provided after weaning can lead to cognitive impairment associated with spatial memory, learning and predisposing to develop Alzheimer disease in younger ages; (2) to examine if early life social experience has long term effects on behavior and cognitive responses and risk for development Alzheimer disease in later life and (3) To determine if an increase of social interactions in adult degu reared in different degree of social stressful conditions alter their behavior and cognitive responses.

## Background

Cognitive ecology focuses on the effects of information processing and decision making on animal evolutionary fitness [1, 2]. A case in point is social behavior. Indeed, studies of social behavior comprise a broad spectrum of interactions among conspecifics that result in variable relationships form, duration, and function [3, 4]. A fundamental aspect of social behavior that arises from social interactions among individuals is the tendency for conspecifics to live in groups. Group living among mammalian species denotes a number of individuals living and interacting together [5, 6], and can occur in from short-term associations and aggregations (e.g., foraging or roosting groups) to relatively long-term socially cohesive units (e.g., communally rearing groups) [7, 8].

Evolutionary explanations to group living have relied on fitness advantages to group members including an increased access to resources, decreased predation risk, decreased burrowing costs, reduced cost of thermoregulation or even increased access to mates [5, 8, 9]. On the other hand, the evolution of group living itself has been attributed to the development of remarkable cognitive capacities [10, 11]. Some of these higher cognitive mechanisms are individual recognition of conspecifics, understanding of their behavioral signals, learning and monitoring of social hierarchies [11].

On the contrary, group living also may impose net fitness cost, leading inevitably to a conflict of interests between group members (e.g., competition for access to limited resources such mates or food, increased parasitism) or on ecological constraints that might force individuals to form groups despite the associated costs [12–14].

These adaptive and nonadaptive scenarios can vary in space and time in response to ecological factors [15, 16]. Thus, studying intraspecific comparisons of mammalian sociality in populations inhabiting different environments remains a major, ultimate explanation of the evolutionary basis of sociality [17, 18]. However, this variation has not revealed a consistent relationship between ecological variation and group living [19–21] suggesting that these mechanisms are not sufficient to explain sociability.

Recent advances in neuroscience, endocrinology, and molecular genetics offer the opportunity to incorporate predictions for how these factors upon which selection can act to shape social systems and allows understand proximate mechanisms of social behavior still in an ecological context [4, 22].

The relation between these internal mechanism and social behavior is bidirectional (i.e., social behavior and its variation in social systems can affect physiological and neuroendocrine mechanism) [23, 24]. Therefore, this new approach offers opportunities to integrate ultimate level function and proximate level mechanism to explore social behavior and gain a comprehensive and integrative understanding of these relationships and also predict the fitness consequences (thus, evolutionary significance) of social systems.

### Social interaction and health

Social interactions appear to have a strong effect on the hypothalamic–pituitary–adrenal (HPA) axis activity [25, 26]. The HPA axis has been regarded as the body´s primary stress response [27]. Nevertheless, recent researches have proposed that activation of HPA system can have consequences that may or may not be linked to responses to stressors [28, 29]. Then, depending on the circumstances, the social relationships between animals that form stable social units or live in close proximity to conspecifics, could be regarded as a source of stress or, alternatively provide a buffer against stress [26, 27]. For example, group living species present a high intraspecific degree of flexibility in social structure, even within group members [16, 30]. If well many species are characterized for establish stable affiliative bonds, and the category of partner effectively acts as a social buffering calming another group member [27]. There also circumstances under social partner can represent a source of stress increasing HPA responses [26, 31]. Lastly, social relationships where a dominance structured or social hierarchies system are established, the level of stress associated with being a dominant versus subordinate animal varies across species and may be related to the behavioral styles of the dominant animals and the level of social stability [26].

#### Stressful live events

The deleterious effects of stress on the immune system are well established in animal and human studies [32, 33]. In fact, stress is an inevitable aspect of living being’s span life. The term stress has been defined as a biological response elicited when an individual face with unpredictable and life threatening perturbations in the environment [34, 35]. These threats elicit physiological (e.g., HPA system and sympathetic nervous system) and behavioral (e.g., fight or flight or enhanced fear or anxiety) responses [36, 37]. Then, an organism wills response to a hostile situation depending not only on type, quality, intensity and duration of stressor, but also on how past experiences and available coping options style its perception of the stressful stimulus [38, 39]. Stress can be moderate and beneficial (e.g., stressful stimuli can play an adaptive role in preparing an animal for coping with later environmental conditions), or it can be long lasting [36, 39, 40]. The prolonged and/or exaggerated exposure to stress initiates a cascade of cells signaling events that would culminate in cognitive disorders, immunosuppression, metabolic syndrome, diabetes, osteoporosis, reproductive failure, and hypertension [39, 41]. In the brain, excess of steroid hormones secretion is strongly associated with neuronal atrophy and dysfunction, and impaired cognition, as well as mood and affective disorders such as depression [37, 39].

Variation in environmental factors such as photoperiod, temperature, food availability, the environment in which an animal is raised and/or housed, or the individual dominance status and social interactions (or lack thereof) can lead to chronically elevated HPA axis activity and a deterioration of health [35, 40, 42]. For example, nonhuman primates and other species housed in unstable social groups (by periodic reorganization of group memberships) exhibit more agonistic encounters and disrupted patterns of affiliative interactions, and ultimately survive a shorter time period compared to animals housed in stable social groups [42–44]. In addition, the social status of group members and its instability (e.g., death, immigration, or emigration of a key individual, or the formation of a new group) appears to be a major source of physical and psychological stress [40, 42, 45]. Furthermore, in those mammal species (even humans) that leave their natal group and move to a nearly o new group, the immigration period may be stressful for both the immigrating and the members of the group that he is joining [26, 45, 46]. Evidence from human and nonhuman animals studies exposing to early life adversity (e.g., maternal separation or social isolation from conspecifics) profoundly affects brain development displaying several long lasting changes in behavior contributing to the prevalence of physical and psychological disorders in adulthood [47–49].

Furthermore, research now indicates that the effects of stress at different period of life interact, meaning that exposure to stress early in life can increases reactivity to stress and cognitive impairments in adulthood [50]. Alternatively, the instability of the social environment in which the pregnant and lactating female lives is another stressful experience for fetal brain development and the behavioral profile of the offspring in later life [51]. Studies reported that mothers subjected an unstable social environment brings a behavioral and neuroendocrine masculinisation in daughters and a less pronounced expression of male typical traits in sons [51–53].

#### Social interactions as buffering

In highly social animals (rodents, birds, nonhuman primates and also in humans) the ability of a social partner to reduce stress responses is commonly referred to as “social buffering” [27, 38, 54]. Many of the benefits achieved through social bonding are thought to result from suppressed HPA axis activity [25, 27, 55], and also has positive effects on the sympathetic nervous system and the immune system responses [27, 38, 56].

Social buffering of stress responses has been extensively studied in the context of mother infant bonding. Across a number of mammalian species the mothers and infants appear strongly attached emotionally, suggesting that the presence of the mother inhibit the infant’s HPA axis; further, infants can buffer the response of mothers [27, 57, 58]. The importance of social buffering also have been documented in intermediate stages of development, and in adulthood of a number of mammalian as well as avian species (Table 1 in Ref. [27]), in particular the presence of familiar social partners and/or salient social relationships. Moreover, in humans, social interactions also appears to have a profoundly influence on human welfare and health, improved diagnosis and treatment several neuropsychiatric disorders [38, 59, 60], and also decreasing mortality from different causes [26, 61]. For instance, disruptions of social relationships could result in behaviors similar to those found in human depression [4, 62, 63], anxiety and also was associated with abnormal physiologic responses as cardiac disturbances [64].

#### Social interactions and aging

Aging is a progressive functional decline, as such, characterized not only by a gradual deterioration of physiological function, including a decrease in fecundity [65, 66], but also by a variety o changes in anatomy, endocrine systems, neural circuitry, as well as behavior [67, 68]. Due to these changes, ageing represents a period of high vulnerability to unstable or adverse environmental conditions, which could accelerate cognitive impairments and hippocampal dysfunction [50, 69]. In fact, increased HPA activity with age, and the resulting elevations of stress related hormones have been linked with hippocampal degeneration (i.e., atrophy and ultimately death of hippocampal neurons with a posterior decreased hippocampal volume) and occurrence of severe cognitive impairments and memory deficit [50, 69, 70].

In socially living individuals this cognitive impairment was associated with disruptions in social motivations and the ability to maintain social relationships primarily due to problems in the recognition and identification of sensory cues used by conspecifics [71–73]. The cognitive ability to memorizing and recalling past actions by conspecifics, know their social relation, predicting their future actions, and adjusting its own behavior in response are critical for the structure and stability [11, 71, 73]. If with increasing age, some of these cognitive abilities decline, then animals may have exhibit aggressive defensive unconditioned reflexes, a decrease in the frequency and quality of social contact leading to social isolation, and ultimately develop stress related disease, such a depression or anxiety [71, 74–76].

#### Stress, aging and Alzheimer’s disease

There is extensive evidence about the association between stress, aging process and their causal role in the development of neuro and psychopatologies such Alzheimer’s disease (AD) [39, 77]. For example, stressful events during lifespan on an individual hasten the appearance of certain biological markers of brain aging that accelerate the onset and progression of AD [39, 77].

The AD is the most common of the brain degeneration [78]. It also was attribute as a primarily form of dementia in the elderly (accounting for up to 70 % of dementia cases) characterized by progressive memory loss and neuropathological changes in specific regions of the brain with deadly outcome [79, 80]. The major pathological hallmarks of AD brains are the massive neuronal cell and synapse loss matter at specific sites and the accumulation of a significant numbers of neurofilament tangles (NFT) and neuritic plaques primarily in the hippocampus, cortex and other brain areas linked to cognitive processes [80–82]. NFT consist of intracellular twisted nerve cell fibers composed of hyperphosphorylated tau, a low molecular weight microtubule associated protein [81]. Whereas plaques are primarily composed of β amyloid (Aβ). Aβ is a short peptide that is an abnormal proteolytic by product of the transmembrane protein amyloid precursor protein (APP), whose function is unclear but thought to be involved in neuronal development [81–83]. There is substantial evidence to show that these NFTs and amyloid plaques and their distribution in the brain correlate with cognitive dysfunction [84, 85].

The clinical characteristics of AD engage progressive impairment or disturbance of multiple brain functions, including memory, orientation, attention, learning capacity, language (aphasia), recognizing or identifying objects (i.e., agnosia), and motor activity (i.e., apraxia) [83, 86]. Unfortunately, the definitive diagnosis method for AD can only be obtained postmortem examinations of brain tissues [87, 88]. A combination of brain imaging and clinical assessment questions for signs of memory impairment have been used to identify patients with AD and other dementias [79, 87].

### Mechanisms of “risk factors” for AD

The average age of diagnosis of AD in humans is around 50 years, with a progressive increase in incidence with increasing age. In fact nearly 50 % of individuals over the age of 85 is affected with this pathology [83, 89]. If well age itself is the single most important risk factor for sporadic AD, the development of this pathogenesis is multifactorial, with genetic, environmental and lifestyle factors implicated [83, 90]. There is an AD that runs in family history of dementia, primarily in those with early onset AD compared with those with late onset [83, 91, 92]. This familial form of AD is due to alterations in three specific genes: presenilin-1 (PS1, on chromosome 14), presenilin-2 (PS2, on chromosome 1) and amyloid precursor protein (APP) that can be inherited as an autosomal dominant disorder and accounts for less than 1 % of the total number of AD cases [79, 82, 92].

Gender is another risk factor for AD, being two to three times more common in females than males [92–94]. Female’s cognitive impairments may also be more severe than males [93–95]. These major sex differences in the incidence and age of onset of AD lies in that different hormone enter in the brain at different times [93]. Estrogens are neuroprotective with respect to neuronal degeneration [92, 96]. When estrogens levels drop at menopause the brain volume beings to decline, particularly in the hippocampus and parietal lobe (areas associated with memory and cognition) [92, 94, 97]. Studies with estrogen replacement therapy showed a delay of 29 % on the onset of AD and even an improve memory in Alzheimer’s patients [93, 96, 98]. On the other way, males are relatively spared because their continuing testosterone secretion is converted, to some extent, to estradiol in the brain (e.g., a men over the age of 60 have three times more circulating estadiol than women of a similar age) [92, 93, 99].

Epidemiological studies have demonstrated the role of environmental factors as diet, activities, or diseases (e.g., type 2 diabetes, hypertension, obesity), psychosocial factors (e.g., depression), as a well history of brain trauma (e.g., cerebrovascular disease, and vasculopathies) to influence both the onset and the progression of AD [83, 100]. For example, due to the high metabolic demand for energy in the brain, small perturbations in glucose metabolism are been expected to affect cognitive performance [79, 101]. Type 2 diabetes (T2DM) has been linked with lower levels of neuronal growth factors, a decreased brain volume and also as an important risk factor for AD development [100, 102]. Lifestyle factors like obesity, poor diet and sedentary behavior, in association with heredity represent the major risk factors for development of insulin resistance, a proximal cause of T2DM [103, 104] and other hypertension, dyslipidemia and cardiovascular disease [86, 105]. There is substantial evidence in animal studies and humans linking diet induced obesity to development and progression of cognitive dysfunction such that higher adiposity means a major risk of developing memory impairment [86, 106]. Furthermore, studies have confirmed association between an increased body mass index with decreased brain volume [107]. Other clinical studies outlined that overweight in humans is associated with reductions in several brain areas involved in the regulation of taste, reward, and behavioral control [108]. Altogether insulin resistance pathology and obesity may lead to much higher incidence and prevalence of AD (86; 104). Other medical conditions that can increase the risk of developing AD include the presence of other disease processes such as Parkinson’s disease, Huntington’s disease, multiple sclerosis and HIV. Down syndrome and some other learning disabilities also increase a person’s risk of dementia [91, 109].

Additional studies suggest that lack of social affiliation (e.g., small social network, participating in small quantitative and low quality of social relations) or social isolation (i.e., physical or contact absence of other members within a social species) has been associated with rapid decline of cognitive function and may contribute to develop AD in late life [110, 111]. Furthermore, investigations of the role of the social environment in health promoting from the stand point of cognitive develop showed that increasing positive social interactions led to improve cognition and buffering against to stressors [112, 113]. For example, animals subject to social isolation developed cognitive impairment and present an early onset and accelerate progression of AD via enhancing activity of certain proteins which plays important role in the production of Aβ peptide and phosphorylation of tau [114, 115]. In humans community, socially isolated individuals have increased risk of developing AD and two to four times increased risk of dead compared with individuals with social ties to friends and relatives [110, 114]. Thus, a high lifelong level of social attachments represents dynamic and complex social systems that affect health outcomes, particularly attaining environmental protection against AD.

Taken together, these data suggest that genetic and environmental influences could be one mechanism behind the wide variation in the onset and progression of AD.

### *Octodon degus,* a model in integrative research of Alzheimer disease

Nonhuman animal research represents an important translational approach to elucidate the mechanistic aspects of the neuropathological characteristics of AD and to validate potential therapeutic targets [116]. There are an important number of nontransgenic animal models (e.g., nonhuman primates, dogs, rabbits, guinea pigs, rats and human) where amyloid deposition increase with age [117–121]. For example, with age, neurodegenerative changes in nontransgenic OXYR rats become amplified, accompanied by accumulation of soluble Aβ, and phosphorylation of the insoluble tau protein, as well as synaptic losses and neural cell death [122].

Additionally, the development of transgenic animal models provides insights to study and understand the molecular mechanisms in AD [116, 123]. To this end, researchers incorporate in these animals human genes known to cause the disease [124] or to perform intracerebral injections of Aβ aggregates that progress with age [125]. However, despite being vital tools, these transgenic animal models have been severely criticized because the development of AD not progresses at the same rate, not always reach the same regions of brain and also the mutated genes are often overexpressed, thus, they are unable to recapitulate all of the pathological features of AD [116, 126, 127].

The native rodent species from central Chile, degu (*Octodon degus*) are particularly appropriate for studying the “natural” development of AD [80, 127]. Aged degus spontaneously develops neuropathological hallmarks of AD, and constituting the first wild-type rodent model for the study of AD neuropathology [127], moreover, there is a high homology (97.5 %) between the human and degu Aβ peptide [127]. Thus, because of this, aged brains of degu (i.e., age 3 and 5) naturally develop accumulation of senile plaques and neurofibrillary tagles [127,128, also see Table 3 in Ref. [116]).

Degus are diurnal, medium sized rodents (ca. 180 g). Degus are socially plural breeding animals, where social group is comprise of 1–5 males and 1–8 multiple lactating females sharing underground nests with communal care of offspring [129–131]. Degu females have a gestation period of about 90 days, giving birth litters of 4–8 pups [132]. Like human babies, degus are born with open eyes, present functional acoustic systems and the pups are capable of detecting even subtle social environmental changes and interact with their littermates and colony mates immediately after birth [132–134]. Despite their maturity at birth, degu pups show close dependence of maternal milk to complete their postnatal development [133, 135]. Although they are able to eat solid food before 6 days of age, the weaning does not occur before than 30 days age [133, 135]. Infant and juvenile degus also show strong social attachments [132]. In fact, stressful factors during the first weeks of life as maternal separation and deprivation of interactions with peers impair a host of neuropsychological and neuroanatomical changes in the brains of young degus [132, 136]. Similar alterations have been found in human’s brain circuits in individuals growing up in adverse environments [132]. Taken together, this highly evolved social organization, which many times recapitulate the richness of human social relationships, degus have been proposed as a good model to study physiological and behavioral traits, including cognitive and sensory abilities [128, 132, 133].

Under laboratory conditions, degu are characterized with a generally docile temper, ease of breeding and maintenance. More notable is the fact that in laboratory environments, degu can live close to 8–10 years’ old, given the observation that between 85 and 95 % of degu under natural conditions do not survive to their second year of age [137]. Age degu (i.e., more than 3 years) spontaneously develop several degenerative disease such as diabetes, atherosclerosis, cancer and Alzheimer’s disease, analogous in many cases to those experimented by humans [127, 128, 138]. Thus, degu constitute an ideal model for biomedical research in general and neuro-ethological studies in particular [132].

### Towards a unifying experimental approach

With the increase of average lifespan of human population, AD is progressing rapidly and has become the major public health problem in the industrialized world. AD patients not only lose their memory and their cognitive abilities, but even their personalities may change dramatically [139]. Scientific community is continually searching new approaches aimed to prevention, delay the onset of symptoms and/or eventually prevent the disease. In this respect, the strong similarity between *O. degus* and human (e.g., lipoprotein metabolism, social organization, cognitive capacities to manipulate objects and learn to use tools) make degu a unique comparative model to identify potential treatment therapies and for assess the complex social behavior at proximate and ultimate levels [126, 140, 141].

**Fig. 1:**
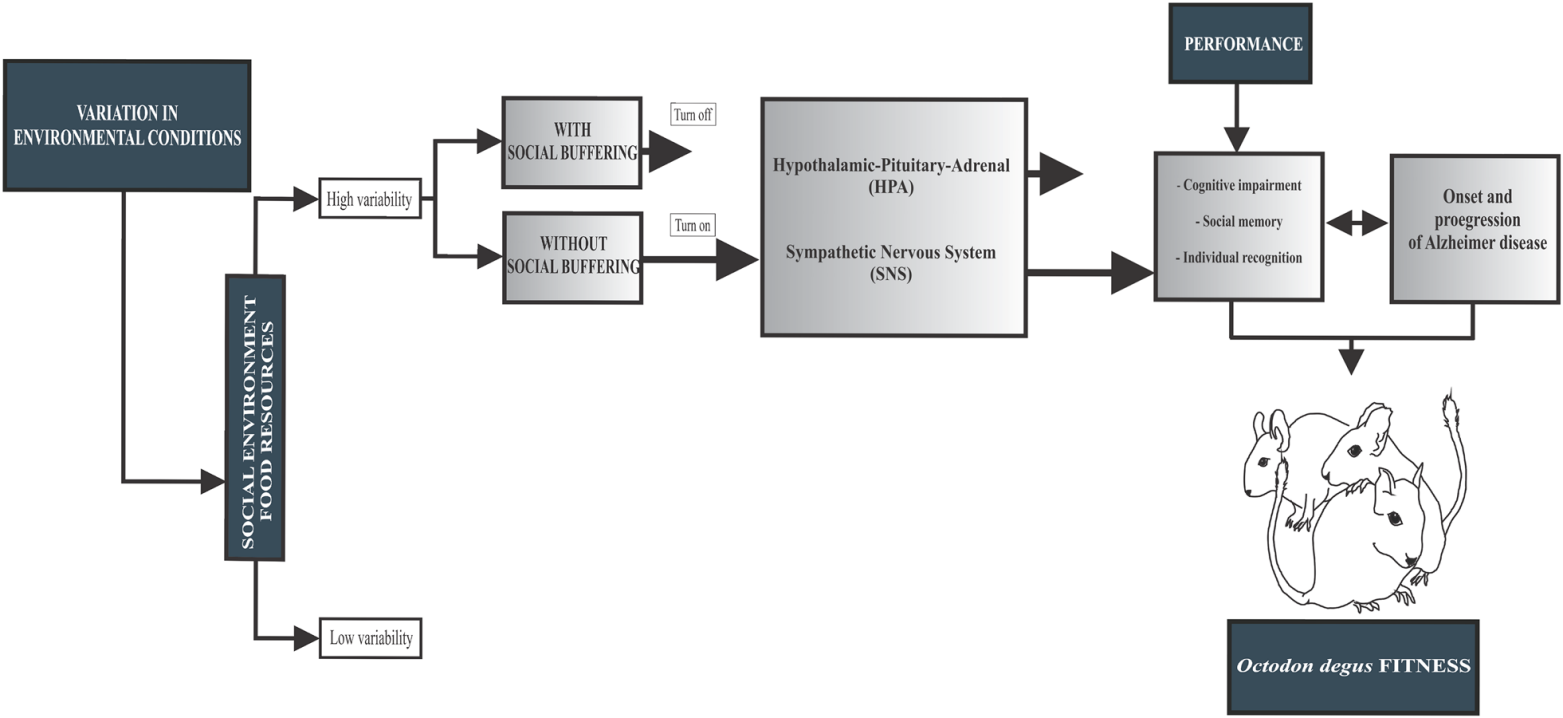
Conceptual model of a program comprising interdisciplinary research proposed in *Octodon degus*. Variation in environmental conditions (e.g., presence or absence of conspecifics, food resources, predators, temperature) can perturb an animal´s homeostasis, and should be act has a potential stressor. Stable social environment in which the presence of the social partners reduce stress responses either before, during, or after stressor exposure to stressors has been associated with control ongoing activity of the HPA of the hypothalamic–pituitary–adrenocortical (HPA) system, which act as the body’s primary stress-responsive neuroendocrine system. Additionally, positive social interactions also have positive effects on other physiological responses, particularly those of the sympathetic nervous system (SNS). Whereas instable social bonding or social isolation during infancy have the opposite effects, which in turn produce more frequent activation of the HPA and SNS systems. The increase in this endocrine activity was associated with more rapid cognitive impairment associated with learning, memory processes and brain structures involved in social behavior in particular those associated with social bonding. Positive social interactions can partially ameliorate this brain injury, and has positive effects in health. Then, social interactions or lack thereof, superimposed to aging process, can determine a progressive amyloid formation and deposition, and neurofibrillary tangles in the brain and may start process associated to AD. Understanding how *Octodon degus* perceive and process their sensory environment under different factors superimposed upon the aging process, is vital to understand whether brain aging is successful or unsuccessful, and examines the disease states

Moreover, degus have become an important model to test how variation in environmental factors (e.g., diet and social behavior) in the context of social interactions or lack thereof, superimposed to aging process, can determine amyloid formation and deposition, and neurofibrillary tangles in the brain and may start process associated to AD (see Fig. 1). Understanding how this animal species perceive and process their sensory environment under different factors superimposed upon the aging process, is vital to understand whether brain aging is successful or unsuccessful, and examines the disease states. We therefore suggest neuroecological approaches to examine how key elements of the environment may affect neural and cognitive mechanisms associated with learning, memory processes and brain structures involved in social behavior in particular those associated with social bonding. We therefore summarize the following three core objectives of a program comprising interdisciplinary research in degus (Fig. 1):

1. To determine whether diet types provided after weaning can lead to cognitive impairment associated with spatial memory, learning and predisposing to develop AD in younger ages. For instance, since *O. degus* spontaneously can develop diabetes, and diabetes is one of the major risk factor for AD development, a high sugar diet can lead to development of diabetes during the first years of life and similarly increase the risk for AD in younger ages.
2. To examine if early life social experience (i.e., stressful social environment) has long term effects on behavior and cognitive responses and risk for development AD in later life (infancy, adolescence, adulthood or aging).
3. To determine if an increase of social interactions in adult degu reared in different degree of social stressful conditions alter their behavior and cognitive responses. For instance an increase in social interaction can help to re socialize behaviorally disturbed degus and mitigate the effects of stress experimented during early life, ameliorating some pathological features as cognitive impairment such as decrease in learning and memory processes.

## Conclusions

Summarizing, cognitive ecologist posits that animal cognition is a biological trait that has been molded by natural selection, thus, the more efficiently an animal uses information from the biotic and abiotic environment, the more adaptive are its cognitive abilities. Nevertheless, this approach does not test for natural neurodegenerative processes under field or experimental conditions, which may improve animals information processing and decision making and may explain, mechanistically, maladaptive behaviors. Overall, we call for integrative approaches to explain the relationship between ultimate (e.g. group stability) and proximate (e.g., cognitive processes) mechanisms behind social behavior. We also emphasize the importance of using the endemic *O. degus* as a valuable natural model for mechanistic studies of social behavior and to explain how physical environments can shape social experiences that might influence impaired cognitive abilities and the onset and progression of neurodegenerative disorders.
